# Long-term exposure of *Aedes aegypti* to *Bacillus thuringiensis* svar. *israelensis* did not involve altered susceptibility to this microbial larvicide or to other control agents

**DOI:** 10.1186/s13071-018-3246-1

**Published:** 2018-12-29

**Authors:** Karine da Silva Carvalho, Mônica Maria Crespo, Ana Paula Araújo, Renata Santana da Silva, Maria Alice Varjal de Melo-Santos, Cláudia Maria Fontes de Oliveira, Maria Helena Neves Lobo Silva-Filha

**Affiliations:** Department of Entomology, Instituto Aggeu Magalhães-FIOCRUZ, Recife, PE 50740-465 Brazil

**Keywords:** Cry11Aa, Cry4Ba, Temephos, Diflubenzuron, Detoxifying enzymes, Resistance

## Abstract

**Background:**

*Bacillus thuringiensis* svar*. israelensis* (Bti) is an effective and safe biolarvicide to control *Aedes aegypti*. Its mode of action based on four protoxins disfavors resistance; however, control in endemic areas that display high mosquito infestation throughout the year requires continuous larvicide applications, which imposes a strong selection pressure. Therefore, this study aimed to investigate the effects of an intensive Bti exposure on an *Ae. aegypti* strain (RecBti), regarding its susceptibility to Bti and two of its protoxins tested individually, to other control agents temephos and diflubenzuron, and its profile of detoxifying enzymes.

**Methods:**

The RecBti strain was established using a large egg sample (10,000) from Recife city (Brazil) and more than 290,000 larvae were subjected to Bti throughout 30 generations. Larvae susceptibility to larvicides and the activity of detoxifying enzymes were determined by bioassays and catalytic assays, respectively. The Rockefeller strain was the reference used for these evaluations.

**Results:**

Bti exposure yielded an average of 74% mortality at each generation. Larvae assessed in seven time points throughout the 30 generations were susceptible to Bti crystal (resistance ratio RR ≤ 2.8) and to its individual toxins Cry11Aa and Cry4Ba (RR ≤ 4.1). Early signs of altered susceptibility to Cry11Aa were detected in the last evaluations, suggesting that this toxin was a marker of the selection pressure imposed. RecBti larvae were also susceptible (RR ≤ 1.6) to the other control agents, temephos and diflubenzuron. The activity of the detoxifying enzymes α- and β-esterases, glutathione-S-transferases and mixed-function oxidases was classified as unaltered in larvae from two generations (F_19_ and F_25_), except for a β-esterases increase in F_25_.

**Conclusions:**

Prolonged exposure of *Ae. aegypti* larvae to Bti did not evolve into resistance to the crystal, and no cross-resistance with temephos and diflubenzuron were recorded, which supports their sustainable use with Bti for integrated control practices. The unaltered activity of most detoxifying enzymes suggests that they might not play a major role in the metabolism of Bti toxins, therefore resistance by this mechanism is unlikely to occur. This study also highlights the need to establish suitable criteria to classify the status of larval susceptibility/resistance.

**Electronic supplementary material:**

The online version of this article (10.1186/s13071-018-3246-1) contains supplementary material, which is available to authorized users.

## Background

*Aedes aegypti* acts as a vector of arboviruses that impact public health worldwide, and Brazil has been dramatically affected by the burden caused by such diseases. Four serotypes of dengue virus are endemic throughout the country [1, 2], in addition to the recent emergence of the chikungunya and Zika viruses [3]. The National Dengue Control Programme, which was implemented in 1996, has been faced with the challenge of controlling *Aedes* [4]; one of the most important issues is the resistance to the organophosphate temephos, a larvicide that has been widely used throughout this programme [5–9]. The use of this compound in several countries has also been reduced due to environmental and safety issues [10].

*Bacillus thuringiensis* svar*. israelensis* (Bti) is one of the most suitable larvicides available for controlling *Aedes aegypti*, whose effectiveness has been demonstrated in outstanding control programmes worldwide [11–15]. The major active ingredients of this microbial larvicide are crystals containing protoxins that display high toxicity to Diptera larvae from genera such as *Aedes*, *Culex*, *Anopheles* and *Simulium*. One of the most remarkable features of Bti is the complex composition of the crystal, which contains protoxins belonging to the 3-domain Cry toxin family (Cry11Aa, Cry4Ba and Cry4Aa) and the cytolytic toxin (Cyt1Aa) [16, 17]. These protoxins act in synergy, as the individual components exhibit lower toxicity than the whole set of components from the crystal, which displays the highest larvicidal action [18]. The major steps of Bti’s mode of action include the ingestion of the crystals by larvae, their solubilization in the midgut and the release of the protoxins that are proteolytically converted into toxins. Subsequently, these activated toxins interact with midgut receptors, insert in the membranes provoking pore formation that leads to cell permeability and osmotic lyses that damage the epithelium [19]. Different models have been proposed explain their mode of action. In the sequential binding model of Cry toxins to receptors [20, 21] the Cyt1Aa has a central role because it has the ability to act as receptor for the Cry toxins, as was demonstrated for Cry11Aa and Cry4Ba [22, 23]. In this model, the monomeric forms of the activated Cry toxins bind to midgut-bound cadherins, or to the Cyt1Aa toxin; this binding promotes the formation of oligomeric forms and these bind with high affinity to other midgut-bound receptors, which can be glycosylphosphatidylinositol-anchored proteins such as aminopeptidases, alkaline phosphatases and maltases [24, 25]. Under the oligomeric state, these toxins are able to insert their domain I in the cell membranes to form pores [26]. Another model, the signaling pathway, proposes that the monomeric activated toxins bind specifically to the cadherins receptors and activates an Mg^2+^ dependent and adenylyl cyclase/protein kinase A signaling pathway that leads to necrotic cell death [27]. Some authors have argued that both models might occur [28], and further data concerning these models and their limitations are discussed by Vachon et al. [19].

This unique role of Cyt1Aa acting as receptor for the other crystal components [29] is a key element for the low potential of resistance selection to Bti, despite its utilization in control programmes for more than three decades. Indeed, consistent cases of resistance have not been recorded in field populations or laboratory strains when larvae were exposed to the whole Bti crystal [30], as will be further discussed. Conversely, selection using single Bti toxins, such as Cry11Aa, Cry4Aa and Cry4Ba, can lead to resistance, as demonstrated by Georghiou & Wirth [31] and supported by subsequent studies [32–37]. In general, the physiological and molecular mechanisms that larvae might display in response to exposure to Bti toxins under continuous selection pressure have been scarcely investigated. For instance, some studies have claimed that Bti exposure could be associated with an increase in the activity of detoxifying enzymes [38–40], which are responsible for provoking metabolic resistance to some chemical insecticides. These enzymes can metabolize certain insecticidal compounds and prevent their access to receptors or target sites, and their role in the resistance to synthetic insecticides, including temephos, has been extensively demonstrated [41, 42].

The spread of *Aedes* spp. worldwide, in addition to their sustained permanence in the territories already occupied [43–46], indicates the need to exploit control agents and strategies that have been adapted to local conditions where the control programmes will be implemented [47]. Control actions in tropical endemic regions, where mosquitos proliferate intensively throughout the year, involve a higher frequency of treatments and, consequently, an increased selection pressure caused by larvicides utilization, compared to control operations in temperate regions, which are carried out only during a few months of the year. Long-term experiences of Bti utilization for *Aedes* control in tropical countries are still scarce, and there is a growing increase of Bti utilization, not only for the conventional treatment of breeding sites but also for its use in association with innovative approaches, such as in inundative lethal ovitraps [48, 49] and spatial spraying in urban areas [50, 51]. Thus, the major goal of this study was to evaluate the effects of prolonged Bti exposure in an *Ae. aegypti* strain on its susceptibility to Bti and other insecticidal compounds (temephos/diflubenzuron), in addition to the respective profile of detoxifying enzymes. The long-term effects of Bti exposure need to be investigated to foster the rational utilization of Bti within integrated control actions.

## Methods

### *Aedes aegypti* strains

Two strains maintained at the insectary of IAM-FIOCRUZ were used in this study: Rockefeller is an international insecticide susceptibility standard, kindly provided by the Laboratório da Superintendência de Controle de Endemias (SUCEN, Marília-SP, Brazil), that has been maintained in the insectary since 2007; RecBti is the test strain of this study and was established with eggs collected in the neighborhoods of the Recife metropolitan region and exposed to Bti, as described below. All strains were maintained at 26 ± 1 °C and 70% humidity, with a 14:10 h light:dark photoperiod. Larvae were reared in dechlorinated tap water and fed with cat food (Friskies®). Adults were fed with a sucrose solution (10%) and females were also artificially fed on defibrinated rabbit blood once per week.

### Control agents

Microbial Bti-based larvicides, the organophosphate (OP) temephos and the insect growth regulator (IGR) diflubenzuron were tested. The Bti samples used in this study are described below. Bti crystals were from the IPS-82 lyophilized reference powder (Instituto Pasteur, Paris, France) containing crystals/spores from sporulated whole culture (H14 serotype) with 15,000 International Toxic Units (ITU)/mg. Cry11Aa and Cry4Ba lyophilized powders (IAM-FIOCRUZ) containing crystals/spores of each recombinant protoxin were obtained from a sporulated culture of the Bti 4Q2-82 acrystaliferous strain. This strain was transformed with the pHT640 or pHT618 plasmids containing *cry11Aa* and *cry4Ba* coding genes, respectively. Bacterial growth and procedures to obtain the lyophilized powders were previously described [52]. These protoxins were chosen to be tested against *Ae. aegypti* larvae because they display the highest activity among the protoxins from the Bti crystal. VectoBac® WG (Valent BioSciences, Illinois, USA) is a commercial product presented as water dispersible granules containing 37.4% of crystals/spores of Bti H-14 (AM6552 strain) with a potency of 3000 ITU/mg. Susceptibility to temephos and diflubenzuron was tested using technical powders with 95.6% (Sigma-Aldrich #31526, St. Louis, USA) and 99.5% (Sigma-Aldrich #34174) of the active ingredient, respectively. Bioassay procedures are described below.

### Establishment of the RecBti strain

The parental strain was established using approximately 10,000 eggs collected in ovitraps set in 40 of the 94 neighborhoods of Recife city. This strain was maintained for 30 generations with exposure to Bti at every generation. For this purpose, samples of the third-instar larvae of each generation were treated with VectoBac® WG (0.5 mg/l) to provide approximately 50% mortality after a 24 h exposure period; this concentration was determined in preliminary tests. In each generation, several batches of 400 larvae were placed in receptacles with dechlorinated water (1 l) and were treated for 24 h. After this period, the larval mortality was recorded and the survivors were then transferred to another receptacle, with water and food and were reared to adulthood to form the subsequent generation. In this study, samples of at least 5000 larvae per generation were exposed, and at least 1000 adults that survived after the Bti exposure were obtained to compose the subsequent generation. The larval susceptibility to the different compounds tested was investigated to assess individuals from different generations during the course of the study.

### Bioassays

The susceptibility of the RecBti larvae to the control agents tested was evaluated using larvae from the Rockefeller strain as the standard reference. The activity of Bti, two of the individual Bti protoxins (Cry11Aa/Cry4Ba) and temephos was determined based on their lethal concentrations (LC) for 50% (LC_50_) and 90% (LC_90_) of larvae after a 24 h exposure period, as previously described [53, 54]. In summary, groups of 20 third-instar larvae, uniformly selected, were placed in 100 ml tap water and treated with a range of five to eight concentrations using serial dilutions from a stock sample of test compounds. The range of concentrations used, in order to provide a response between 10–100% mortality after exposure, was determined in preliminary tests. Stock suspensions of lyophilized powders containing a mixture of Bti or Cry spores-crystals in water (5 g/l) and a stock solution of temephos in ethanol (3 g/l) were prepared and stored at -20 °C until use. Untreated groups with water (for Bti/Cry toxins) or solvent/water (for temephos) were run in each test. Data from the bioassays whose mortality in the untreated control groups was higher than 10% were not employed to establish the LCs. All treated and untreated groups were tested in triplicate in each bioassay, and these were performed on at least three different dates. Lethal concentrations were determined through Probit analysis (SPSS 16.0 for Windows). Resistance ratios (RR) between the LC to the RecBti strain and the respective LC to the reference strain were provided. For temephos, RRs between 3- and 5-fold, between 5- and 10-fold, and above 10-fold were classified as low, moderate, and high resistance, respectively, according to Mazzari & Georghiou [53]. For Bti/Cry toxins, RR values lower than 10-fold were considered to be natural variations based on the dataset of Bti susceptibility of untreated populations available in the literature. The effect of the IGR diflubenzuron was determined by the emergence inhibitory concentrations (EIC) for 50 and 90% of adults from the larvae samples that were exposed to this compound, according to the procedures described by Martins et al. [55]. For these bioassays, six replicates of groups formed by ten third-instar larvae, uniformly selected, were treated with a range of five to seven concentrations using serial dilutions of a stock solution that had been prepared in acetone (3 mg/l) and stored at -20 °C. Those larvae were kept exposed to diflubenzuron for a 30 day period under laboratory conditions with a regular provision of food, as described for the maintenance of the laboratory strains. Emerging adults and dead larvae and/or pupae from all groups were recorded and removed from the receptacles every day. The assays were performed at least three times on different dates.

### Enzymatic assays

The catalytic activity of esterases (α-EST, ß-EST), glutathione-S-transferases (GST), and mixed function oxidases (MFO) was determined in RecBti and Rockefeller strain larvae. The specific activity of each enzyme group was measured in individual third-instar larvae according to the protocol of Ministério da Saúde [56], which was adapted for larvae. For this purpose, individual larvae previously stored at -70 °C were macerated in 100 μl water (MilliQ® Millipore, Billerica, USA) and then centrifuged (425× *g*, 4 °C). Supernatants were collected and kept at 4 °C and then the aliquots were immediately used to determine the protein content [57] and the respective catalytic activity [56]. Enzymes activity was evaluated with different substrates: α-naphthyl acetate (Sigma-Aldrich #N8505) to α-EST; β-naphthyl acetate (Sigma-Aldrich #N6875) for ß-EST; 1-chloro-2,4-dinitrobenzene (CDNB, Sigma-Aldrich #C-6396) and reduced glutathione (GSH, Sigma-Aldrich #G6529) for GST; and 3,3’,5,5’-tetramethyl-benzidine dihydrochloride (TMBZ, Sigma-Aldrich #T8768) for MFO. Independent batches of approximately 100 larvae from each strain were used to test the activity of each class of enzymes assessed. First, the 99th percentile of the activity recorded for the larvae of the Rockefeller strain was established, which was then used as a reference to classify the activity of RecBti larvae. These were classified according to the frequency of individuals who display an activity that is higher than the 99th percentile of the Rockefeller strain larvae as follows: unaltered (U) for less than 15% of larvae; altered (A) for between 15 and 50% larvae; and highly altered (HA) when more than 50% of larvae is above that parameter [9].

## Results

### Selection of the RecBti strain

In the course of this study, third-instar larvae from this strain were exposed to a Bti-based product (VectoBac® WG) for 30 generations, as summarized in Additional file 1: Table S1. At every generation, a mean of 9535 larvae, varying between 5700 and 21,600, were exposed to Bti for 24 h. During the selection process, a total of 292,550 larvae were exposed and only those individuals who survived the Bti treatment to reach the adult phase were employed to compose the subsequent generations. Larval mortality showed variations after 24 h exposure, but individuals from all generations were subjected to levels that were close to or higher than 50%. An additional mortality of around 15% was recorded after the 24 h Bti exposure until the adult phase. In some generations, when the mortality after treatment was much higher than 50%, as detected in F_6_ and F_10_, a greater number of larvae was subjected to treatment in order to always ensure a large adult sample to compose the next generation. During the selection process, the final mortality varied between 63–91%, with an average of 74%. A mean of 2322 adults was obtained per generation from the initial sample of Bti-treated larvae. Overall, the selection of RecBti strain involved samples with a large numbers of individuals from diversified origin from Recife city which were intensively exposed to Bti under controlled conditions to ensure the selection pressure.

### Susceptibility to Bti and its protoxins

**Fig. 1:**
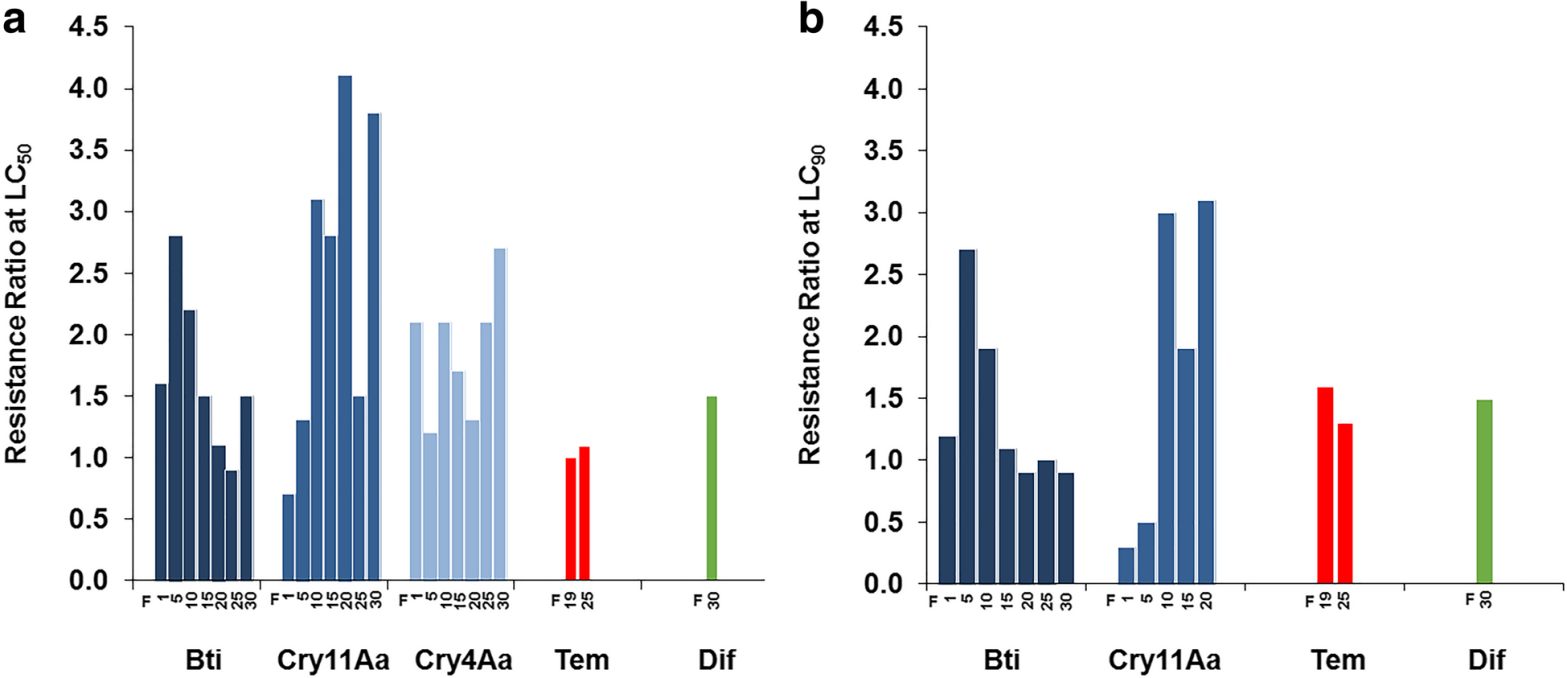
Resistance ratios (RR) between the lethal concentrations of Bti and its toxins (Cry11Aa, Cry4Ba), temephos (Tem) and diflubenzuron (Dif) for third-instar *Ae. aegypti* larvae from the RecBti strain compared to that of the reference strain. **a** RR at LC_50_. **b** RR at LC_90_

The susceptibility of RecBti larvae to different compounds was investigated with an emphasis on Bti and its protoxins, which were assessed at every five generations from F_1_-F_2_ to F_30_ in order to detect potential alterations in the course of the selection process (Table 1). Bioassay data from all generations assessed showed that RecBti larvae displayed a similar susceptibility to Bti, according to the LC_50_ and LC_90_ values, compared to that of the Rockefeller reference larvae (Fig. 1). These LCs were close to the reference strain, and the highest resistance ratios detected, 2.8 at LC_50_ and 2.7 at LC_90_, were both recorded in F_5_. There was no increase of these LCs in subsequent evaluations, and only slight variations were detected. The Bti LCs towards F_30_ larvae, which were subjected to the longest exposure period, were similar to those detected for the reference strain (RR_50_ = 1.5 and RR_90_ = 0.9, respectively).

**Table 1:** Toxicity of *Bacillus thuringiensis* svar. *israelensis* (IPS-82) and its protoxins to Bti-selected *Ae. aegypti* larvae

Sample^a^	No. of larvae	LC_50_ (95% CI)^b^	RR^c^	LC_90_ (95% CI)^b^	RR^c^
Bti					
Rocke	1620	0.008 (0.007–0.009)	1.0	0.026 (0.021–0.036)	1.0
RecBti					
F_1_	1480	0.013 (0.011–0.016)	1.6	0.030 (0.024–0.048)	1.2
F_5_	1380	0.022 (0.018–0.028)	2.8	0.069 (0.048–0.143)	2.7
F_10_	1080	0.018 (0.015–0.020)	2.3	0.049 (0.039–0.070)	1.9
F_15_	1380	0.012 (0.010–0.013)	1.5	0.029 (0.024–0.038)	1.1
F_20_	1680	0.009 (0.007–0.010)	1.1	0.024 (0.021–0.030)	0.9
F_25_	1680	0.007 (0.006–0.009)	0.9	0.026 (0.022–0.035)	1.0
F_30_	1680	0.012 (0.010–0.013)	1.5	0.024 (0.022–0.029)	0.9
Cry11Aa					
Rocke	1080	0.162 (0.121–0.210)	1.0	1.506 (0.909–3.525)	1.0
Rocke^d^	1260	0.436 (0.338–0.666)	1.0	2.470 (1.650–8.576)	1.0
RecBti					
F_2_	1600	0.121 (0.099–0.145)	0.7	0.512 (0.396–0.727)	0.3
F_5_	1440	0.217 (0.183–0.256)	1.3	0.754 (0.571–1.052)	0.5
F_10_	1440	0.498 (0.369–0.642)	3.1	4.515 (2.717–12.965)	3.0
F_15_	1260	0.459 (0.365–0.568)	2.8	2.923 (2.020–5.116)	1.9
F_20_	1260	0.672 (0.540–0.846)	4.1	4.677 (2.965–9.055)	3.1
F_25_^d^	1260	0.653 (0.472–0.924)	1.5	nd	nd
F_30_^d^	2400	1.662 (1.203–2.400)	3.8	nd	nd
Cry4Ba					
Rocke	1080	0.331 (0.209–0.492)	1.0	nd	nd
RecBti					
F_2_	1280	0.694 (0.483–0.978)	2.1	nd	nd
F_5_	1200	0.402 (0.304–0.508)	1.2	nd	nd
F_10_	1200	0.707 (0.435–1.265)	2.1	nd	nd
F_15_	2100	0.570 (0.396–0.806)	1.7	nd	nd
F_20_	1200	0.424 (0.276–0.610)	1.3	nd	nd
F_25_	1260	0.701 (0.487–0.989)	2.1	nd	nd
F_30_	1260	0.882 (0.575–1.426)	2.7	nd	nd

*Abbreviation*: *nd* not determined

^a^Rockefeller (Rocke) reference strain and the RecBti selected strain

^b^Lethal concentrations (mg/l) for 50% and 90% of third-instar larvae exposed for 24 h (mean and 95% confidence intervals, CI)

^c^Resistance ratio between the LC for the test strain and that for the reference strain

^d^Reference used to determine RR for F_25_ and F_30_

The activity of the Cry11Aa and Cry4Ba protoxins to larvae was evaluated since these are the Bti crystal components that exhibit higher individual toxicity to larvae, although still lower than that of the whole crystal (Table 1). Cry11Aa toxicity first evaluated towards the larvae of the reference Rockefeller strain at LC_50_ was approximately 20-fold lower than that of the Bti LC towards this same strain. The respective LCs of Cry11Aa to RecBti larvae were slightly higher than that for the larvae of the Rockefeller strain in all generations assessed. In the evaluations from F_2_ to F_20_, the RR_50_ values varied between 0.7–4.1-fold, compared to those of the Rockefeller reference strain. An LC increase of Cry11Aa for RecBti larvae was observed in the next assessments at F_25_ and F_30_, and a novel evaluation of the Rockefeller larvae was performed in order to compare these LCs in equivalent conditions. This second evaluation of Cry11Aa toxicity towards larvae of the Rockefeller strain yielded an RR at LC_50_ for the RecBti of 1.5 and 3.8-fold in F_25_ and F_30_ larvae, respectively. The RR value the F_30_, which was the last evaluation performed, suggested a tendency for an increase at LC_50_, considering the values found in last time points analyzed. In addition the LC_90_ in these evaluations was not achieved in the range of concentrations tested. These data suggest that a reduction of Cry11Aa toxicity towards RecBti larvae might be under-way, even if the RR values at LC_50_ are still lower than 10-fold. The toxicity of Cry4Ba for Rockefeller larvae was approximately 41-fold lower than that of the Bti crystal. For Cry4Ba, only LC_50_ values were determined, since this toxin alone did not achieve high mortality levels in order to establish confident values of LC_90_. The susceptibility of RecBti to Cry4Ba displayed discrete variations of a similar range (RR ≤ 2.7) in most assessments performed throughout the study. The activity of Cry4Ba did not reveal any sign of alteration that could be associated with the progression of the selection process imposed on the RecBti strain. The resistance ratios that were recorded for Bti and its protoxins throughout the study are shown in Fig. 1.

### Susceptibility to temephos and diflubenzuron

The susceptibility of RecBti larvae to the OP temephos was assessed using larvae from F_19_ and F_25_ generations when a long period of Bti exposure had been already achieved. RecBti larvae from both generations showed similar LCs to those detected for the reference strain, and the RR at LC_50_ and LC_90_ were between 1–1.6-fold (Table 2) in those evaluations. According to the criteria established for temephos [53], these larvae are classified as displaying an unaltered status of susceptibility (RR < 3). The lethal concentrations of temephos for larvae from both strains displayed narrow confidence intervals and indicated that the activity of this compound showed a limited range of variations compared to those that were detected for Bti. The activity of diflubenzuron, which is a chitin inhibitor, was tested towards RecBti larvae from F_30_, and the concentrations that prevented 50 or 90% of adult emergence from the treated RecBti larvae (F_30_) were similar to the reference strain and yield an RR of 1.5-fold (Table 3). These evaluations show that the RecBti larvae exposed to Bti showed susceptibility to all control agents tested (Fig. 1).

**Table 2:** Toxicity of temephos to Bti-selected *Ae. aegypti* larvae

Sample^a^	No. of larvae	LC_50_ (95% CI)^b^	RR^c^	LC_90_ (95% CI)^b^	RR^c^
Rocke	1380	0.007 (0.007–0.008)	1.0	0.010 (0.010–0.011)	1.0
RecBti F_19_	1680	0.007 (0.007–0.008)	1.0	0.016 (0.012–0.018)	1.6
Rocke	1620	0.007 (0.007–0.008)	1.0	0.011 (0.010–0.014)	1.0
RecBti F_25_	1980	0.008 (0.007–0.008)	1.1	0.014 (0.012–0.016)	1.3

^a^Rockefeller (Rocke) reference colony and the RecBti selected strain (generations F_19_ and F_25_)

^b^Lethal concentrations (mg/l) for 50% and 90% of third-instar larvae exposed for 24 h (mean and 95% confidence intervals, CI)

^c^Resistance ratio between the LC for the test strain and that for the reference strain

**Table 3:** Activity of diflubenzuron to Bti-selected *Ae. aegypti* larvae

Sample^a^	No. of larvae	EIC_50_ (95% CI)^b^	RR^c^	EIC_90_ (95% CI)^b^	RR^c^
Rocke	1680	0.774 (0.662–0.903)	1.0	1.422 (1.226–1.940)	1.0
RecBti F_30_	1680	1.188 (1.050–1.320)	1.5	2.123 (1.911–2.416)	1.5

^a^Rockefeller (Rocke) reference strain and the RecBti selected strain (generation F_30_)

^b^Emergence inhibitory concentrations (μg/l) for 50% and 90% of adults from third-instar larvae exposed for 30 days (mean and 95% confidence intervals, CI)

^c^Resistance ratio between the EIC for the test strain and that for the reference strain

### Activity of detoxifying enzymes

The activity of four classes of enzymes mainly associated with insecticide metabolism was evaluated in larvae from RecBti and Rockefeller strains. The activity of α-esterases (α-EST), β-esterases (β-EST), glutathione-S-transferases (GST) and mixed function oxidases (MFO) was assessed in larvae from F_19_ and F_25_ of the RecBti strain, in parallel with the temephos evaluation previously described. These activities were compared with those detected for Rockefeller reference larvae and were assessed at each evaluation carried out (Table 4). RecBti larvae from F_19_ displayed a level activity of all four classes of enzymes, which was similar to the respective activity detected in Rockefeller larvae, since the frequency of individuals whose activity was above the 99th percentile of the Rockefeller was absent or low (0–11%) for all of the enzymes assessed. In the second evaluation conducted after 25 generations of Bti exposure, the larvae showed an activity profile for all enzymes that was similar to that of the Rockefeller strain, except for the β-esterases, which displayed an altered status of 27% of individuals tested whose activity was higher than that of the reference.

**Table 4:** Activity of detoxifying enzymes from Bti-selected *Ae. aegypti* larvae

Larvae sample^a^	α-EST			β-EST			GST			MFO		
	*n*	> 99%^b^	Status	*n*	> 99%^b^	Status	*n*	> 99%^b^	Status	*n*	> 99%^b^	Status
Rockefeller	150	–	–	89	–	–	96	–	–	112	–	–
RecBti F_19_	159	0	U	86	2	U	121	2	U	99	11	U
Rockefeller	167	–	–	128	–	–	142	–	–	96	–	–
RecBti F_25_	160	2	U	159	27	A	113	2	U	111	0	U

*Abbreviations*: *α-EST* α-esterases, *β-EST* β-esterases, *GST* glutathione-S-transferases, *MFO* mixed-function oxidases, *U*, unaltered for percentage below 15%, *A*, altered for percentage between 15% and 50%

^a^Third-instar larvae from the Rockefeller (Rocke) reference strain and the RecBti selected strain from generations F_19_ and F_25_

^b^Percentage of individuals whose activity is higher than the 99th percentile of the Rockefeller larvae

## Discussion

The *Ae. aegypti* RecBti strain from this study subjected to treatments with a Bti-based larvicide during 30 generations kept full susceptibility to this agent. Data show the lack of resistance selection to the whole Bti crystal and corroborates previous attempts using *Ae. aegypti* and *Culex pipiens* complex strains in the laboratory [31, 35, 58–62], as well as in Bti treated-populations [5, 63–67]. In this regard, it is important to notice that the single potential record of Bti resistance of *Cx. quinquefasciatus*-treated populations [68] did not provide consistent evidence to directly associate this finding with the use of Bti. The present study promoted suitable conditions of selection, which included the large and diversified larvae samples that were subjected to a strong and sustained selection using a Bti-based commercial product containing whole crystals. It is worth noting that under field conditions, climatic (e.g. rainfall, solar radiation) and/or operational (e.g. closed houses, low frequency of treatments) factors can limit the range of larvicide application and thus reduce selection pressure, contrarily to the selection performed under controlled laboratory conditions. In our study, RecBti larvae did not display altered susceptibility to Bti, showing that the risk of selection associated with the use of this compound is effectively low.

On the other hand, previous studies have shown that strains subjected to selection with the whole Bti crystal did not display resistance to Bti but could display resistance to the single toxic components [34, 35, 37] and, in this study, the susceptibility to two individual Cry protoxins from Bti was also assessed. Cry11Aa and Cry4Ba displayed decreased activity in larvae from the reference strain compared to that of the whole crystal, which reinforces that synergism among all Bti toxins is needed to achieve its highest toxicity level and complexity of action, as previously shown [18]. The susceptibility of RecBti larvae to Cry4Ba did not show significant alterations during the selection process, while the signs of reduced susceptibility recorded for Cry11Aa between F_25_ and F_30_ seem to reflect the selection pressure imposed on this strain. The monitoring of the Cry11Aa and Cry4Ba in other Bti-selected strains showed higher RR alterations, which were between 6–16-fold after 18–30 generations, and the greatest levels of 30-fold and 68-fold were recorded for the Cry4Aa [34, 35, 62]. In our study, the susceptibility to Cry4Aa was not assessed since it exhibits the lowest activity among Bti toxins [18], leading to wide variations in the LCs, and monitoring the susceptibility to Cry11Aa was more sensitive to indicate that the strain was under selection pressure. The genetic background of mosquito samples and selection procedures are also variables that can play a role in the responses to these toxins during the selection process. For instance, the Bti sample used in this study were crystals from a commercial larvicide (37.4% of crystal/spores as active ingredient), while some studies have employed indirect source of Bti, as Bti components found in soil leaf litter [37]. These toxic litter samples contain recycled or persistent Bti protoxins but the proportion and amount of Cyt1Aa was much lower compared to that found in Bti crystals [37], and this is critical considering the crucial role of Cyt1Aa for the synergy of the Bti toxins. Under the conditions of our study, the selection pressure was directly imposed by the use of larvicides containing the Bti protoxins, in the expected rates, which were applied in the aquatic environments with *Aedes* larvae. This could be a factor explaining the higher resistance ratios observed to the individual toxins amongst the studies. Despite these variations, our study corroborates others that have shown that the monitoring of single protoxins can be a useful tool for demonstrating the response of larvae under selection pressure with Bti, even if the susceptibility to the Bti whole crystal is not disrupted [69]. Related to these findings, it also worth noticing that alterations to the susceptibility to single toxins, *per se*, do not have implications in terms of Bti effectiveness, as the commercial larvicides used in interventions contain whole crystals with a full set of protoxins at the expected ratio that can overcome the potential failures of single Cry components [23, 29]. In this scope, a study has shown, for instance, that larvae from *Ae. aegypti* strains selected with individual protoxins showed failures in toxin receptors while a strain selected with the whole Bti crystal did not show alterations in these molecules [70].

The occurrence of potential cross-resistance patterns of the Bti-selected larvae to other control agents was also relevant for evaluation, since they are frequently employed in *Aedes* control programmes. RecBti larvae were fully susceptible to temephos, and data corroborate previous reports in which temephos-resistant populations, some of which displayed high resistance levels, were susceptible to Bti [5]. Diflubenzuron had a similar performance to inhibit adult emergence from RecBti and the reference-treated larvae. Cross-resistance to these compounds, specifically due to target site alterations, was not expected, since organophosphates are neurotoxins that act in the acetylcholinesterase [41] and diflubenzuron disrupts the molt process acting through the chitin synthase [71]. However, these compounds could display cross-resistance due to the metabolic mechanism caused by detoxifying enzymes, which had been already recorded for some conventional chemical compounds [42], and is under investigation for the IGR pyriproxyfen [72] and the biological larvicide Bti [38, 40, 73]. This aspect will be further discussed in the scope of the detoxifying enzymes profile that was found in RecBti larvae. Further, in regard to the comparative toxicity detected for compounds performed in this study, narrower confidence intervals for temephos and diflubenzuron LCs were found compared to those detected for Bti. One reason for that could be with the route of action of compounds, since chemicals act directly by contact and bacterial insecticidal crystals act by ingestion. This latter mode of action depends on physiological factors, for instance feeding rates according to the developmental stages and food availability. In addition, chemical compounds are uniformly synthesized at high purity levels while microbial compounds are products from biological processes that are subjected to more variation, and the protein nature of the insecticidal crystals makes them more vulnerable to degradation [74, 75].

Bti-selected strains that exhibit the RR of 2- or 3-fold, for instance, have been previously reported as resistant [37, 76], although susceptibility surveys have shown that populations that have not been treated with Bti displayed resistance ratios in the range of 4-fold for *Aedes* [5, 73, 77–79] or 12-fold for *Cx. pipiens* [80]. Such variations, regardless of a previous exposure to this larvicide, can naturally occur. Therefore, data from our study and from the literature indicate that the criteria for classifying the status of susceptibility/resistance to a certain compound should take into account the baseline data available for the mosquito samples. Monitoring the susceptibility of untreated field populations is essential to build a baseline and to establish suitable criteria, and to avoid the use of only laboratory strains as a reference for susceptibility.

The mechanism of metabolic resistance to the conventional chemical insecticides associated with the increased activity of detoxifying enzymes has been extensively reported [42], while their role in the metabolism of insecticidal bacterial toxins remains obscure. The mode of action and targets of Bti toxins and neurotoxins from chemical compounds are distinct, as described before. Data from our study demonstrated that Bti-selection was not correlated with a marked increase in the activity of detoxifying enzymes in Bti-exposed larvae and no alteration in the susceptibility to the insecticidal compounds tested was detected. On the other hand, the association between the increased activity of detoxifying enzymes and the historic of temephos exposure has been described in several studies. A previous survey, for instance, showed that twelve temephos-resistant *Ae. aegypti* populations (7 < RR < 253) and a high-resistant laboratory-selected strain (RR ~180) had increased activities of least three of the five classes of detoxifying enzymes that were assessed [5]. A transcriptomic profile of two *Ae. aegypti* strains, one selected with Bti and another with chemical insecticides (pyrethroid, neonicotinoid and carbamate), showed that gene coding for such enzymes were under- and overregulated, respectively [81]. This scenario suggests that the detoxifying enzymes may not have a major role in the metabolism of such bacterial toxins, although additional studies to investigate this aspect are still needed.

## Conclusions

The RecBti strain exposed to Bti crystals did not display a loss of susceptibility to Bti or to two of its protoxins after 30 generations of intensive selection pressure with a Bti-based larvicide. Data reinforce that, to date, there is no consistent record of resistance to the whole crystal, although monitoring of Cry11Aa toxicity indicated that this strain was under selection pressure. RecBti larvae were also susceptible to the organophosphate temephos and to the IGR diflubenzuron, which shows that these agents can be used along with Bti in integrated actions. The exposure to bacterial Bti was not associated with a marked increase in detoxifying enzymes, which suggests that cross-resistance by this pathway is unlikely to occur. The dataset from this study validates the use of Bti associated with innovative methods to effectively control to *Aedes* spp. with a low risk of resistance selection.

## Additional file

Additional file 1: **Table S1.** Summary of the exposure of third-instar *Ae. aegypti* larvae from the RecBti strain with *Bacillus thuringiensis* svar. i*sraelensis* (VectoBac® WG), under laboratory conditions. (DOCX 15 kb)

## Abbreviations

95% CI: 95% confidence interval
Bti: *Bacillus thuringiensis* svar. *israelensis*
CNDB: 1-Chloro-2,4-dinitrobenzene
EIC: Emergence inhibitory concentration
GST: Glutathione-S-transferases
GST: Reduced gluthatione
IGR: Insect growth regulator
ITU: International toxic units
LC: Lethal concentration
MFO: Mixed function oxidases
OP: Organophosphates
RR: Resistance ratio
ß-EST: ß-esterase
TMBZ: 3,3’,5,5’-tetramethyl-benzidine dihydrochloride
α-EST: α-esterase
