# Supplementary material for: Long-term exposure of Aedes aegypti to Bacillus thuringiensis svar. israelensis did not involve altered susceptibility to this microbial larvicide or to other control agents
Source: Parasit Vectors. 2018 Dec 29;11:673. doi: 10.1186/s13071-018-3246-1 (PMC6311009; doi:10.1186/s13071-018-3246-1)
Supplement: Supplementary file 1 — Table S1. Summary of the exposure of third-instar Ae. aegypti larvae from the RecBti strain with Bacillus thuringiensis svar. israelensis (VectoBac® WG), under laboratory conditions. (DOCX 15 kb) [file 13071_2018_3246_MOESM1_ESM.docx]

**Table S1.** Exposure of Ae. aegypti larvae to Bacillus thuringiensis svar. israelensis under laboratory conditions

| **Generation** | **No. of larvae^a^** | **Mortality 24 h (%)** | **Mortality^b^ (%)** | **No. of adults** |
| --- | --- | --- | --- | --- |
| Parental^c^ | 11,600 | nd | 74 | 3000 |
| F_1_ | 8350 | nd | 69 | 2573 |
| F_2_ | 5700 | nd | 79 | 1209 |
| F_3_ | 12,000 | nd | 77 | 2702 |
| F_4_ | 7800 | nd | 63 | 3082 |
| F_5_ | 8100 | 58 | 69 | 2531 |
| F_6_ | 17,700 | 75 | 85 | 2590 |
| F_7_ | 11,100 | 50 | 76 | 2682 |
| F_8_ | 8700 | 53 | 69 | 2691 |
| F_9_ | 10,200 | 57 | 72 | 2506 |
| F_10_ | 14,700 | 80 | 91 | 1322 |
| F_11_ | 6400 | 59 | 63 | 2530 |
| F_12_ | 10,500 | 59 | 84 | 3440 |
| F_13_ | 12,900 | 65 | 88 | 1557 |
| F_14_ | 11,200 | 64 | 85 | 1717 |
| F_15_ | 21,600 | 66 | 76 | 5182 |
| F_16_ | 11,600 | 60 | 69 | 3570 |
| F_17_ | 9600 | 65 | 75 | 2412 |
| F_18_ | 9200 | 59 | 71 | 2617 |
| F_19_ | 6400 | 65 | 74 | 1828 |
| F_20_ | 9600 | 57 | 68 | 3023 |
| F_21_ | 7200 | 66 | 74 | 1812 |
| F_22_ | 6800 | 59 | 73 | 1836 |
| F_23_ | 7200 | 58 | 72 | 1982 |
| F_24_ | 6800 | 56 | 75 | 1099 |
| F_25_ | 6000 | 51 | 79 | 1252 |
| F_26_ | 6000 | 52 | 76 | 1651 |
| F_27_ | 6400 | 47 | 66 | 2162 |
| F_28_ | 6800 | 64 | 79 | 1430 |
| F_29_ | 8000 | 45 | 66 | 2723 |
| F_30_ | 6400 | 45 | 65 | 1938 |
| Mean | 9535 | 59 | 74 | 2322 |

^a^ Third-instar larvae from the RecBti strain treated with VectoBac^®^ WG (0.5 mg/l)

^b^ Final mortality based on the number of survivors who reached the adult stage

^c^ Parental generation established with eggs collected in 40 neighborhoods of Recife city

*Abbreviations*: nd, not determined
